# Supplementary material for: State of lifestyle medicine education in Saudi medical schools: A descriptive study
Source: PLoS One. 2024 Aug 8;19(8):e0308499. doi: 10.1371/journal.pone.0308499 (PMC11309438; doi:10.1371/journal.pone.0308499)
Supplement: Attachment — Submitted filename: report.docx [file pone.0308499.s001.docx]

**Reviewer report for PLOS one Journal**

Title: State of lifestyle medicine education in Saudi medical schools

1. **Abstract:**
   1.1. In abstract, method section, the statement: (We aimed

to involve at least one administrative position holder from each of these 38 medicals

colleges in this study to gain an accurate understanding of the current curriculum.) is not clear.

1.2. The result section in abstract is deficient, it is not supported by enough findings.
1.3. The authors mentioned in result section that (All three course specific areas (nutrition, physical activity, and health & wellness coaching) were mostly evaluated by written assessments) This finding is not supported by number or percentage.
1.4. In abstract, result section, the authors wrote (MCQs, OSCE, Essay, SEQ/SAQ, Assignments, and OSPE were the assessment techniques of the LM domains). This statement is belong to methodology section not a result.

2. **Introduction:** suitable and expressive.

The objectives are clear, and so the presentation

3. **Presentation and methods:** the methods are well described.
4. **Results section:**

This part is good but I suggest the following:

4.1. The author should add a regression table to demonstrate the factors that influence the deficiency in awareness of LM among the participants.

5. **Interpretation:**  satisfied
6. **Conclusion:** satisfied
