# Supplementary material for: State of lifestyle medicine education in Saudi medical schools: A descriptive study
Source: PLoS One. 2024 Aug 8;19(8):e0308499. doi: 10.1371/journal.pone.0308499 (PMC11309438; doi:10.1371/journal.pone.0308499)
Supplement: Attachment — Submitted filename: response to reviewers comments.docx [file pone.0308499.s002.docx]

**Response to Reviewer Comments**

Dear Editor and reviewers,

Thank you very much for taking the time to review this manuscript. Please find the detailed responses below and the corresponding revisions/corrections highlighted/in track changes in the re-submitted files.

| **Comments** | **Response** |
| --- | --- |
| Editorial comments |  |
| In the online submission form, you indicated that [The datasets generated during and/or analysed during the current study are available from the corresponding author upon reasonable request.].  All PLOS journals now require all data underlying the findings described in their manuscript to be freely available to other researchers, either 1. In a public repository, 2. Within the manuscript itself, or 3. Uploaded as supplementary information. This policy applies to all data except where public deposition would breach compliance with the protocol approved by your research ethics board. If your data cannot be made publicly available for ethical or legal reasons (e.g., public availability would compromise patient privacy), please explain your reasons on resubmission and your exemption request will be escalated for approval | The data generated for the current study is already present in the study in terms of frequency and percentage. Thank you for pointing out our mistake, we have also corrected the statement in the manuscript. |
| Your ethics statement should only appear in the Methods section of your manuscript. If your ethics statement is written in any section besides the Methods, please move it to the Methods section and delete it from any other section. Please ensure that your ethics statement is included in your manuscript, as the ethics statement entered into the online submission form will not be published alongside your manuscript. | We have made the changes as per the journal style and now ethical statement is in the method section. |
| Reviewer 1 | Thank you for your thorough review and valuable feedback. We have made several revisions to address your comments and improve the manuscript. Below are our responses to each of your points: |
| Reviewer #1: 1. Abstract: 1.1. In abstract, method section, the statement: (We aimed to involve at least one administrative position holder from each of these 38 medicals colleges in this study to gain an accurate understanding of the current curriculum.) is not clear. | We have made the suggested changes. |
| 1.2. The result section in abstract is deficient, it is not supported by enough findings. | Thank you for the suggestion. We have made the specified changes in abstract result section. |
| 1.3. The authors mentioned in result section that (All three course specific areas (nutrition, physical activity, and health & wellness coaching) were mostly evaluated by written assessments) This finding is not supported by number or percentage. | We have made the specified changes. |
| 1.4. In abstract, result section, the authors wrote (MCQs, OSCE, Essay, SEQ/SAQ, Assignments, and OSPE were the assessment techniques of the LM domains). This statement is belong to methodology section not a result. | Thank you for pointing this out. The authors appreciate this comment. It is added in the conclusion in the revised version of the manuscript |
| 2. Introduction: suitable and expressive.  The objectives are clear, and so the presentation | We appreciate your positive feedback on the introduction. We are glad that you found it suitable and expressive. |
| 3. Presentation and methods: the methods are well described. | Thank you for acknowledging that the methods are well described. We have ensured that they are detailed and clear to facilitate replication and understanding. |
| 4. Results section: This part is good but I suggest the following: 4.1. The author should add a regression table to demonstrate the factors that influence the deficiency in awareness of LM among the participants. | Our analysis was designed to be simple and descriptive, as the primary objective of our study was to explore the general status of Lifestyle Medicine (LM) teaching across medical colleges in Saudi Arabia. We aimed to provide an overview rather than conducting detailed comparisons of the characteristics of each college.  - Given this objective, we have not included a regression table in the results section. Instead, we focused on presenting descriptive statistics to provide a comprehensive picture of the current state of LM teaching. |
| Reviewer #2: | Thank you for your thorough review and valuable feedback. We have made several revisions to address your comments and improve the manuscript. Below are our responses to each of your points: |
| The English language needs to be revised by either a professional English editing agency or a native speaker. | We have revised the manuscript for clarity and readability. The manuscript has been reviewed by a professional English editing agency to ensure proper language usage and grammatical accuracy. The certificate of English editing has been uploaded as supplementary file. |
| Avoid using pronouns such as "we". | The manuscript has been edited to avoid the use of pronouns such as "we." Statements have been rephrased to maintain an objective tone. |
| The statistics lacks any sign for bivariate analysis. | Our study aimed to provide a descriptive overview of the current state of LM teaching rather than conducting detailed statistical comparisons. Therefore, bivariate analysis was not included in the original design. However, we have clarified this in the manuscript to ensure the study's scope and objectives are clear. |
| No limitations stated or future studies. | A section on limitations and suggestions for future studies has been added to the manuscript. |
| Your conclusion can be shortened and focus on the outcomes. | The conclusion has been shortened and now focuses more directly on the key outcomes of the study. The revised conclusion emphasizes the primary findings and their implications for the future direction of LM education in Saudi medical schools. |

We appreciate your constructive comments and believe that these revisions have significantly improved the manuscript. Please let us know if there are any further adjustments or clarifications needed.

Thank you once again for your valuable feedback.

Best regards,
